# Overcoming Immunological Challenges Limiting Capsid-Mediated Gene Therapy With Machine Learning

**DOI:** 10.3389/fimmu.2021.674021

**Published:** 2021-04-27

**Authors:** Anna Z. Wec, Kathy S. Lin, Jamie C. Kwasnieski, Sam Sinai, Jeff Gerold, Eric D. Kelsic

**Affiliations:** ^1^ Applied Biology, Dyno Therapeutics Inc, Cambridge, MA, United States; ^2^ Data Science, Dyno Therapeutics Inc, Cambridge, MA, United States

**Keywords:** gene therapy, protein engineering, immune evasion, machine learning, AAV capsid design

## Abstract

A key hurdle to making adeno-associated virus (AAV) capsid mediated gene therapy broadly beneficial to all patients is overcoming pre-existing and therapy-induced immune responses to these vectors. Recent advances in high-throughput DNA synthesis, multiplexing and sequencing technologies have accelerated engineering of improved capsid properties such as production yield, packaging efficiency, biodistribution and transduction efficiency. Here we outline how machine learning, advances in viral immunology, and high-throughput measurements can enable engineering of a new generation of de-immunized capsids beyond the antigenic landscape of natural AAVs, towards expanding the therapeutic reach of gene therapy.

## Introduction

Recently approved AAV-based therapeutics and numerous therapeutic candidates in advanced clinical development (1) have demonstrated the transformative and life-saving potential of viral capsids as vectors for gene therapy (GT). The demands on viral capsids to deliver gene replacement and gene editing tools will continue to increase as our understanding of genetic diseases reveals new therapeutic opportunities. Development of next generation capsids that enable more precise, efficient, and durable gene delivery will be key to improving the effectiveness and safety of such therapies. In this perspective, we explore how high throughput (HT) measurement and characterization methods can be combined with machine learning (ML) approaches to identify such capsids by efficiently optimizing capsid sequences for both improved transduction and reduced immunogenicity. Combining these technologies will generate capsid-mediated gene therapies with broader therapeutic uses that are accessible to all individuals in need.

## The Need to Optimize Natural AAV Capsids for Therapeutic Delivery

Most recombinant AAV capsids used clinically today are closely related, or even identical, to naturally occurring AAVs in their amino acid sequences and biological properties. As natural selection did not optimize such capsids for therapeutic use, they display limited specificity of cell targeting and low overall *in vivo* transduction efficiency in many target tissues, particularly following intravenous administration. Improving *in vivo* transduction of target cells and organs would enable gene therapies to more effectively treat diseases, to perdure, and to address new therapeutic applications. Importantly, pre-existing humoral and cellular immunity against natural AAV capsids limits patient eligibility for therapies as well as their therapeutic efficacy (2). Furthermore, capsids possess inherent immunogenicity — the propensity to activate immune responses — which can impact safety and efficacy, as well as the potential for redose. The challenges of evading both pre-existing immunity and *de novo* adaptive immune responses against AAV vectors are made especially difficult by the heterogeneous nature of patient immune responses and immune histories. Thus, discovering capsids that circumvent the immune system is a significant hurdle facing developers of next generation GT vectors (2).

**Figure 1:**
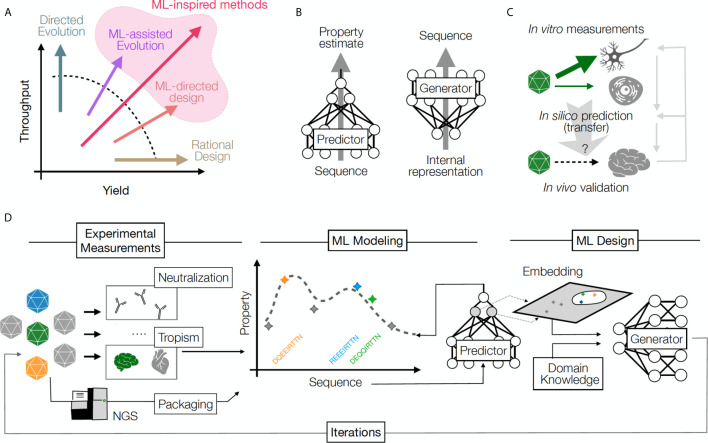
**(A)** A comparison of throughput (number of samples) and yield (fraction of successful samples generated per attempt) for multiple protein design approaches. Rational design increases yield, directed evolution leverages throughput, and ML methods increase the likelihood of success by balancing yield and throughput. **(B)** Predictive ML models map sequences to their functional properties, while Generative methods can turn an internal data representation back into sequences, producing desirable samples. **(C)** An example of transfer learning whereby a model *transfers* information across cell types and experimental contexts: a model learns based on *in vitro* capsid performance in diverse cell transduction experiments (including neurons), then is applied to predict the result of *in vivo* transduction in the brain neurons, when such experimental data is sparse or missing. Information from *in vivo* validation of the predicted capsid performance is used to refine model performance and understand the relationship between *in vivo* and *in vitro* assays. Right grey arrows illustrate the iterative power of this approach, which refines predictive and generative models over time. **(D)** The design cycle starts with HT screening and measurements of several AAV capsid variant properties. These properties are then used to train predictive models that can impute the property for unseen sequences (predictor model) and can be used to build helpful representations (embeddings), which can then be integrated with auxiliary input (e.g., domain knowledge) to propose a batch of new sequences (generator model). The design process can be repeated in multiple iterations until desired capsids are discovered.

Established approaches for obtaining novel capsids include mining the naturally-occurring sequence diversity of capsids, rational design and directed evolution (3–5). Each methodology has contributed valuable capsids to the available catalog of GT vectors, but limitations related to speed and throughput of discovery persist because the total number of possible capsids far exceeds the capacity of current screening approaches. Directed evolution methods often take advantage of ultra-high diversity generated by random mutagenesis in an attempt to overcome the barrier of low discovery yield (i.e. success per individual design). In contrast, rational design approaches rely on expert knowledge and focus on a higher likelihood of success per design, but are relatively low throughput (and overall low yield) as a result. ML approaches offer a promising new option that may mitigate the trade-off between yield and throughput (**Figure 1A**). ML can be used in combination with these established approaches, or as a stand-alone technique to open new avenues of discovery through high-throughput direct synthesis (6).

The set of desired properties that a capsid should possess in order to be therapeutically transformative can collectively be termed a *capsid profile*, in other words the target of optimization efforts. Capsids that embody every therapeutically desirable property outlined above have eluded discovery despite years of effort. Despite the vast number of possible capsid sequences, it is reasonable to assume capsids which achieve these desired profiles, if they exist, are extremely rare in sequence space (7, 8). Reducing the number of required properties in the context of a particular therapeutic application may increase the chance of finding a candidate capsid, but this may come at the cost of failure in later stages of clinical development. The therapeutic usefulness of a given capsid and our ability to find it are therefore fundamentally in tension. In this perspective, we share how new approaches to immunological data gathering, combined with analysis and design approaches powered by ML, are overcoming this tension towards discovery of capsids that are more therapeutically useful.

## Key Concepts for Applying Machine Learning to Engineer Novel Capsids

Recent advances in ML enable new solutions to problems inherent to designing immune-evasive capsids. ML is a collection of algorithmic approaches that allow for automatic learning. These approaches are capable of learning rules for predicting the outcome of complex processes directly from input data. Larger and richer datasets pose a challenge for traditional methods of rational design but are the environment in which ML methods thrive (9). ML models can be considered mathematical approximators of physical processes we have measured, and oftentimes have yet to understand mechanistically (10–12). In the context of biological design, ML models can replace labor- or resource-intensive experiments with *in silico* screening. With increasing amounts of data, these approximations can become very accurate, and their rapid and cost-effective application enables the identification of biological designs which would not be accessible by experimentation alone. Importantly, mechanistic knowledge need not be wasted in this approach — biological insights can be incorporated into ML architectures in a way that bolsters model robustness, allowing for more accurate models trained by less data. Additionally, ML can simplify how we represent and understand high-dimensional and high-throughput data, allowing us to substantially improve the experiments themselves. Finally, while many mechanistic details of AAV gene therapy remain poorly understood, ML models trained on empirical data that can predict capsid functions are sufficiently useful for engineering better capsids despite the models being agnostic to mechanism, and in some cases querying such models can guide or improve our mechanistic understanding.

Key ML concepts illustrate the potential for this approach to transform capsid engineering. First, ML algorithms can learn arbitrary *sequence-to-function* relationships. These relationships can be learned automatically from large datasets of capsid sequences and their measured properties. A model can predict one or multiple properties at once. For instance, models can be trained to learn the relationship between the capsid sequence and its ability to produce a viable capsid (6) or its tropism to the liver (13). These training schemes, termed *supervised*, require collecting data labels (measurements) of the kind we are intending to predict. However, it is also possible to train models solely based on a set of good examples without additional measurements. For instance, training models on the rapidly growing set of publicly available protein sequences to learn relationships among them has shown promise in protein structure and function prediction (12, 14–17). This type of training is known as *unsupervised*. Both supervised and unsupervised training schemes can yield *predictive models* that output property values given an input sequence, or alternatively *generative models* that produce novel sequences given desirable property values as inputs (**Figure 1B**). It is noteworthy that building models with good generalization ability, i.e. ability to predict accurately on samples far from those in the training data, requires care in experimental design and training schemes. Otherwise, models may *overfit* to the training data available, where they perform well on samples similar to their training data, but unexpectedly poorly in novel settings.

Second, effective machine learning methods often make use of internal *latent representations*, also known as *embeddings*, which attempt to represent the information contained in raw inputs in a way that is more amenable to human understanding. One such simple and widely applied method is principal component analysis (PCA), in which a linear transformation of input data allows for the identification of data elements that contribute most to the variance in the data set. PCA and other more complex non-linear dimensionality reduction methods transform high-dimensional raw input data to a lower-dimensional representation (a latent space) that is easier to interpret, visualize, and optimize (14, 18–21). If these and other methods can be applied to the problem of AAV capsid engineering, AAV variant sequences with similar properties to each other would be close together in latent space after being transformed into their latent representations, even if they are far apart in sequence space. A similar strategy was recently used to predict the emergence of escape mutations in multiple viruses (22).

Finally, modern ML can utilize auxiliary data to make inference about domains where information is sparse, a process known as *transfer learning* (**Figure 1C**) (23, 24). An illustrative conceptual example for this technique in machine vision involves “style-transfer” where particular painting styles are learned from an artist’s work, and can then be applied to any new image, converting the style to that of the original artist (25). This type of learning can be used in many contexts in biology (23, 26). For instance, predictive models around AAV serotypes for which little data is available could be improved by training them on data available from other related serotypes or even a larger set of related proteins. Similarly, population level data for immunity profiles of specific patient groups could be used to reduce the amount of data required to make inferences for individual patients. Along with the ability to integrate information from multiple modalities, transfer learning can rapidly accelerate the application of ML models in areas where data is limited, and open new domains for prediction and design. An example of a ML-driven design pipeline is illustrated in **Figure 1D**. These concepts will be useful for designing immune-evasive capsids, as we explain below.

## Safe and Effective Treatment at Lower Doses

Among all capsid properties that could be improved, increased tissue-specific transduction is key to enabling safe and effective gene therapies. Improving this attribute would allow for a higher proportion of injected capsids to deliver their payloads to the intended cells, reducing the dose needed for effective treatment. This in turn would make treatment safer by reducing activation of the innate immune responses and of B and T cell responses, which increase in magnitude relative to the amount of antigenic stimulus (vector dose) delivered (27).

Making viral vectors safer and more effective will require optimization towards multi-property capsid profiles. However, many capsid properties are intrinsically coupled to one another and efforts to optimize or re-direct any single attribute often result in capsids that fail basic tests of functionality, such as capsid assembly and genome packaging. ML models can greatly reduce the burden of multi-property optimization through *in silico* screening of variants (28), ensuring that optimization toward one property does not break other desired functions (29, 30), shifting the engineering burden away from experimental approaches (28). For instance, four supervised models can be trained to learn sequence-to-function maps between capsid sequences and their ability to (i) transduce the liver, (ii) bypass off-target organs, (iii) evade neutralization, and (iv) produce at high yield. The first model can be used in an *in silico* search for variants with better transduction, and the other models can be used to eliminate sequences proposed by the first model that do not meet the specificity, immune evasion and capsid production requirements. A significant body of work in the interface of ML and biology is focused on algorithms that use such supervised models to optimally design protein sequences (31). Notably, while non-human primates are at present the industry-preferred model for measuring transduction, the ability for ML to integrate diverse sources of information may increase the utility of data from other animal models (including transgenic animals with humanized immune systems), as well as human cell culture models, for predicting transduction patterns in human patients and lead to better rates of clinical translation. Capsids optimized towards a profile of improved and specific transduction, reduced immunogenicity, and production efficiencies equivalent to natural AAV capsids would already be transformative relative to currently available vectors.

## Perduring Gene Therapy

In an ideal therapeutic scenario, a single dose of GT would provide a durable, curative effect throughout a recipient’s lifetime. In practice, this goal has been difficult to realize as therapeutic transgene expression from current vectors decays over time (32). Waning transgene expression can result from silencing of the viral genome through epigenetic mechanisms, from cell division, or from transduced cell death, among other factors. One mechanism underlying the loss of transduced cells observed in a number of clinical studies (33–35) was the induction of cytotoxic CD8^+^ T lymphocyte (CTL) responses against cells presenting capsid antigens, for which immunosuppression is the primary clinically viable remedy.

Engineering capsids that reduce or even eliminate CTL responses will facilitate perduring therapeutic gene expression. Transduced cells process viral capsids through the intracellular proteolytic machinery and present capsid-derived peptides on their surface though the major histocompatibility (MHC) class I molecules (33, 34). CD8^+^ T cells recognize presented peptides *via* their highly specific T cell receptors, which in turn determines cell stimulation, proliferation and cytotoxic activity. CTL activation results in killing of transduced cells as well as generation of immunologic memory that poses a barrier for vector redosing. Unlike B cells, which interact with surface exposed capsid epitopes, T cells can in theory sample the full peptidome of an AAV capsid, including buried capsid sequences that drive assembly or disassembly, and which may be more difficult to alter by conventional engineering approaches. Extensive mapping of CD8^+^ T cell epitopes within AAV capsid proteins and evaluation of their propensity to activate T cell responses would identify the key sequences which must be modified to de-immunize AAV capsids. The large diversity of HLA alleles among people and distinct patterns of peptide presentation and recognition determined by them makes this challenging. While it is currently not possible to exhaustively assess peptide presentation by all variants of MHC class I found in humans, emerging ML methods in peptide presentation and immunogenicity prediction (36, 37) will increase the accuracy of these predictions compared to tools available today. Recently developed strategies of experimental immunopeptidome characterization using mass spectrometry (38, 39) will provide a rich source of data for training such models.

Understanding the determinants of capsid antigen presentation (40) and their effect on CTL activation will provide the foundations for ML models to engineer capsids that evade them. The rules of peptide presentation are shared across the entire proteome based upon an individual patient’s HLA alleles (41). This means that ML models can benefit from all existing datasets that catalog CD8^+^ T cell epitopes and learn general properties that influence which peptides tend to be presented in particular genetic backgrounds (17). Through transfer learning, such general models could be tuned toward more accurate models that predict CD8^+^ T cell epitopes for AAV capsid variants specifically. This would require relatively small amounts of additional data that is specific to AAV capsids and would enable engineering of capsids depleted of T cell-activating peptides. While predictions of MHC class I presentation have advanced significantly, meaningful annotation of peptide immunogenicity that enables more accurate models for immunogenicity prediction will require development of HT functional assays and remains an open challenge for the field of T cell biology.

## Gene Therapy for All: Overcoming Pre-Existing Anti-Capsid Antibodies

A majority of prospective GT recipients have pre-existing antibodies against one or more natural AAV serotypes, often excluding them from treatment (42–44). Pre-existing antibodies accelerate vector clearance, redirect vector biodistribution, and can directly inhibit capsid-mediated cell entry (33). To overcome these activities of antibodies, it is critical to identify capsids that cannot be efficiently bound and neutralized by them – in other words, capsids with surface-exposed sequence and structural features not previously encountered by the adaptive immune response. Altering antibody recognition of capsids in a therapeutically meaningful way is challenging because serum antibody responses are highly diverse and can target the entire capsid surface (45, 46). Antibodies bind both linear and discontinuous epitopes on the capsid exterior surface, sometimes spanning across neighboring capsid subunits, making rational approaches to altering these sites challenging. Moreover, neutralizing antibodies often target capsid regions involved in critical functions such as cell receptor recognition, meaning that mutations which prevent antibody binding can also adversely affect vector transduction (47).

Much remains to be learned about how human antibodies bind to and neutralize capsids, however several technologies now enable high-throughput mapping of antibody responses at the monoclonal level. The study of both serum antibodies and antibodies encoded by memory B cells in donors with recent AAV exposures can reveal key characteristics of human anti-capsid antibody responses and provide a more complete picture of anti-capsid antibody immunity. While serum antibodies are maintained at steady state by long lived plasma cells, the memory B cell repertoire approximates the antibody repertoire that will be mobilized on AAV re-encounter and their characterization is methodologically useful as a means of identifying anti-capsid antibody sequences for in depth functional studies. For example, efforts in the infectious diseases therapeutic space have yielded multiple approaches to fine mapping of *de novo* and memory B cell responses, where hundreds or even thousands of virus-specific antibodies encoded by B cells can now be routinely sequenced, cloned and produced (48). Epitopes of such antibodies can be characterized using HT competition assays (49, 50) and correlations can be derived between binding site location and neutralization activity. Recently developed approaches utilizing cryo-electron microscopy (51, 52) and high resolution, quantitative, proteomics-based approaches (53–55) enable serum antibody specificities to be characterized in unprecedented detail, to inform their identities and their binding sites. These and other studies revealed for a number of pathogens that just one class of antibodies can contribute the majority of neutralizing activity in the serum despite the overall high diversity of antibody responses (56–58). Identifying any dominant human neutralizing antibody types against AAVs would inform the sites where capsid engineering can be most effectively applied.

Data with resolution at the individual antibody level would enable ML models to learn how antibody responses target a particular capsid and how to predict their effect on other (designed) capsids. Models can serve as *in silico* evaluators of capsids before they are administered to patients with pre-existing antibodies based on characterization using the methods described above. Through sequencing of capsid-specific B cells and characterization of serum antibodies, a personal ‘immunological fingerprint’ can be created with the aid of ML models, which could also be used to find general patterns in human anti-capsid antibody responses (59). For instance, unsupervised models can directly learn from genetic data to predict immune profile responses. Supervised models could use patient serum data together with other measurements [e.g. sequencing of immune repertoires (59) or genome scanning antibody profiling (60)] to predict likelihood of therapeutic success, or to help select vector administration options. With such models in hand, panels of antibody-evading AAV capsids could be recommended based on a patients’ pre-existing antibody repertoire to maximize the chance of effective antibody evasion.

Many gaps remain in our understanding of how anti-capsid antibodies can be evaded. Serology studies with naturally occurring AAVs have been useful in defining population-level prevalence of anti-AAV immunity but such bulk-level measurements have had limited value for engineering antibody-evading capsids. Some monoclonal antibodies isolated from mice have been characterized in detail (46, 61) providing important insights about the antigenic sites on AAV capsids targeted by neutralizing antibodies. However, it remains a challenge to generalize these results to human antibody responses, which are encoded by distinct germline genes, are more diverse (62), and are shaped in response to a distinct set of natural AAVs endemic in humans. An in-depth large-scale characterization of human antibodies targeting capsids would facilitate our ability to engineer capsids with maximal therapeutic impact.

One such promising approach would be to measure the activity of serum antibodies against highly diverse libraries of capsid variants using immune human serum samples. Such data would enable ML models to learn the quantitative relationship between AAV capsid sequences and their abilities to evade pre-existing antibodies, and to learn commonalities in anti-capsid antibody responses among people. Similarly, intravenous immunoglobulin (IVIg) preparations containing antibodies from thousands of donors may be useful in such screens for identifying the predominant patterns in human antibody responses. Recent work characterizing B cell and antibody responses to a number of important human pathogens (56, 63–65) reveal common features of antibody responses elicited by a given pathogen across donors. If similar shared antibody types arise against AAV capsids, resurfacing the epitopes they target would allow engineering of capsids that more broadly evade antibody activity, towards the goal of creating *universal capsids* capable of treating all patients.

## Future Directions

ML-powered capsid design and engineering will transform the landscape of GT delivery modalities, however non-capsid improvements are also relevant from an immunological perspective and can also increase therapeutic effectiveness. Reducing the activation of innate immunity by engineering the vector genome (66, 67), co-administration with targeted immune-modulators to induce tolerance toward the vector (68) or depletion of pre-existing anti-capsid antibodies (69) should work in synergy with engineered capsids to pave a path for repeat vector administration, while further increasing the safety and tolerability of next generation GTs.

As we have outlined, ML approaches to engineer improved AAV capsids have multiple applications: enabling gene therapies that are effective in a lower dose regimen, removing capsid peptides which elicit cytotoxic T cell responses thereby leading to longer lasting gene expression, and resurfacing capsid exteriors allowing potentially universal treatment of all patients. While these goals are ambitious and each individually worthy of study, combining all such properties in a single capsid would be transformative for the field. ML approaches will facilitate this goal by incorporating information from diverse experimental systems and improving the efficiency of multi-trait capsid optimization. We are optimistic that safe, efficient, target-specific, non-immunogenic and universal capsids will one day enable gene therapy to reach its full potential by delivering therapeutic DNA to cure, treat and prevent disease and even to improve overall health for all patients. Interdisciplinary collaborations focused on combining HT measurements with ML-powered sequence design algorithms will dramatically accelerate progress towards achieving these goals.

## Data Availability Statement

The original contributions presented in the study are included in the article/supplementary material. Further inquiries can be directed to the corresponding author.

## Author Contributions

AW, KL, JK, SS, JG and EK conceptualized, wrote and edited the manuscript. AW and SS prepared figures. All authors contributed to the article and approved the submitted version.

## Conflict of Interest

AW, KL, JK, SS, JG and EK are employees and shareholders in Dyno Therapeutics Inc.
